# Functional in vivo and in vitro effects of 20q11.21 genetic aberrations on hPSC differentiation

**DOI:** 10.1038/s41598-020-75657-7

**Published:** 2020-10-29

**Authors:** Hye-Yeong Jo, Youngsun Lee, Hongryul Ahn, Hyeong-Jun Han, Ara Kwon, Bo-Young Kim, Hye-Yeong Ha, Sang Cheol Kim, Jung-Hyun Kim, Yong-Ou Kim, Sun Kim, Soo Kyung Koo, Mi-Hyun Park

**Affiliations:** 1 Division of Intractable Diseases Research, Department of Chronic Disease Convergence Research, Korea National Institute of Health, Korea Disease Control and Prevention Agency, Cheongju, Republic of Korea; 2 Interdisciplinary Program in Bioinformatics, Seoul National University, Seoul, Republic of Korea; 3 Division of Data Science, University of Suwon, Hwaseong-si, Gyeonggi-do 18323 South Korea; 4 Division of Healthcare and Artificial Intelligence, Department of Precision Medicine, Korean National Institute of Health, Korea Disease Control and Prevention Agency, Cheongju, 28159 Korea; 5 Department of Computer Science and Engineering, Seoul National University, Seoul, Republic of Korea; 6 Division of Regenerative Medicine Safety Control, Department of Chronic Disease Convergence Research, Korea National Institute of Health, Korea Disease Control and Prevention Agency, 202 Osongsaengmyeing2-ro, Cheongju, South Korea

**Keywords:** Induced pluripotent stem cells, Stem-cell differentiation, Structural variation, Transcriptomics

## Abstract

Human pluripotent stem cells (hPSCs) have promising therapeutic applications due to their infinite capacity for self-renewal and pluripotency. Genomic stability is imperative for the clinical use of hPSCs; however, copy number variation (CNV), especially recurrent CNV at 20q11.21, may contribute genomic instability of hPSCs. Furthermore, the effects of CNVs in hPSCs at the whole-transcriptome scale are poorly understood. This study aimed to examine the functional in vivo and in vitro effects of frequently detected CNVs at 20q11.21 during early-stage differentiation of hPSCs. Comprehensive transcriptome profiling of abnormal hPSCs revealed that the differential gene expression patterns had a negative effect on differentiation potential. Transcriptional heterogeneity identified by single-cell RNA sequencing (scRNA-seq) of embryoid bodies from two different isogenic lines of hPSCs revealed alterations in differentiated cell distributions compared with that of normal cells. RNA-seq analysis of 22 teratomas identified several differentially expressed lineage-specific markers in hPSCs with CNVs, consistent with the histological results of the altered ecto/meso/endodermal ratio due to CNVs. Our results suggest that CNV amplification contributes to cell proliferation, apoptosis, and cell fate specification. This work shows the functional consequences of recurrent genetic abnormalities and thereby provides evidence to support the development of cell-based applications.

## Introduction

Human pluripotent stem cells (hPSCs) are a valuable resource for potential cell therapies, regenerative medicine, drug discovery, and disease modelling^1,2^. For these applications, strict safety guidelines are required to verify that the cells are genetically stable. Genomic instability of hPSCs due to recurrent genomic alterations in hPSCs has been reported^3,4^, and large-scale integrated genomic studies have been used to characterize hPSCs^5,6^. Since hPSCs can undergo alternative fates, such as self-renewal, differentiation, or death, genomic alterations including copy number variations (CNVs) in hPSCs would be occurred during the reprogramming process and over time in culture^7^, or technical culture conditions (e.g., freezing and thawing)^8^. These unexpected genomic aberrations cause culture adaptation or selective advantages that influence the genomic instability of hPSCs^9,10^.

CNVs are a major source of genomic variation. The functional effects of CNVs are comparatively greater than those of single-nucleotide polymorphisms (SNPs), with significant consequences for phenotypic variations^11,12^. For example, recurrent CNV is a risk factor for cancers and developmental delays^13,14^. There have also been reports of CNVs in hPSCs at specific genomic locations^15–17^ and recurring at chromosomes 12, 17, 20, and X^18^. These frequent CNV gains indicate that the genes included in these CNV regions may confer adaptive changes in hPSC lines. In particular, the amplification region on the 20q11.21 region, which includes the *ID1*, *BCL2L1*, and *HM13* genes, is well known; ID1 is a regulator of NANOG^19^, and BCL2L1 is a key gene driving the selective advantage of 20q11.21 amplification^8^, resulting in decreased sensitivity to apoptosis^20,21^.

Here, we present a comprehensive assessment of CNVs in human embryonic stem cells (hESCs) and human-induced pluripotent stem cells (hiPSCs) from the Korea Stem Cell Bank (KSCB; https://kscr.nih.go.kr) using our established pipelines to verify whether these hPSC lines contain aberrations derived from genomic instability. In addition, transcriptomic profiling of hPSCs with CNV gain at 20q11.21 was carried out to clarify the expression changes and functional effects. Furthermore, we performed single-cell transcriptome analysis of embryoid bodies (EBs) of normal and mutant hiPSCs to investigate the impact of CNVs at 20q11.21 on hPSC pluripotency and differentiation in vitro. Spontaneous in vivo differentiation of hPSCs with CNV amplification into teratomas was also assessed at the histological and transcriptomic levels, indicating that specific genomic changes in hPSCs must be verified in cell-based applications in terms of genomic stability.

## Results

### Identification of CNV gain at 20q11.21 in hPSCs

We analysed seven hPSC lines from the KSCB, including hESCs and hiPSCs, at both early and late passages and two primary cell types. Supplementary Fig. S1a and Table S1 present detailed sample information, and the cell lines have been characterized in previous reports^22–24^.

**Figure 1:**
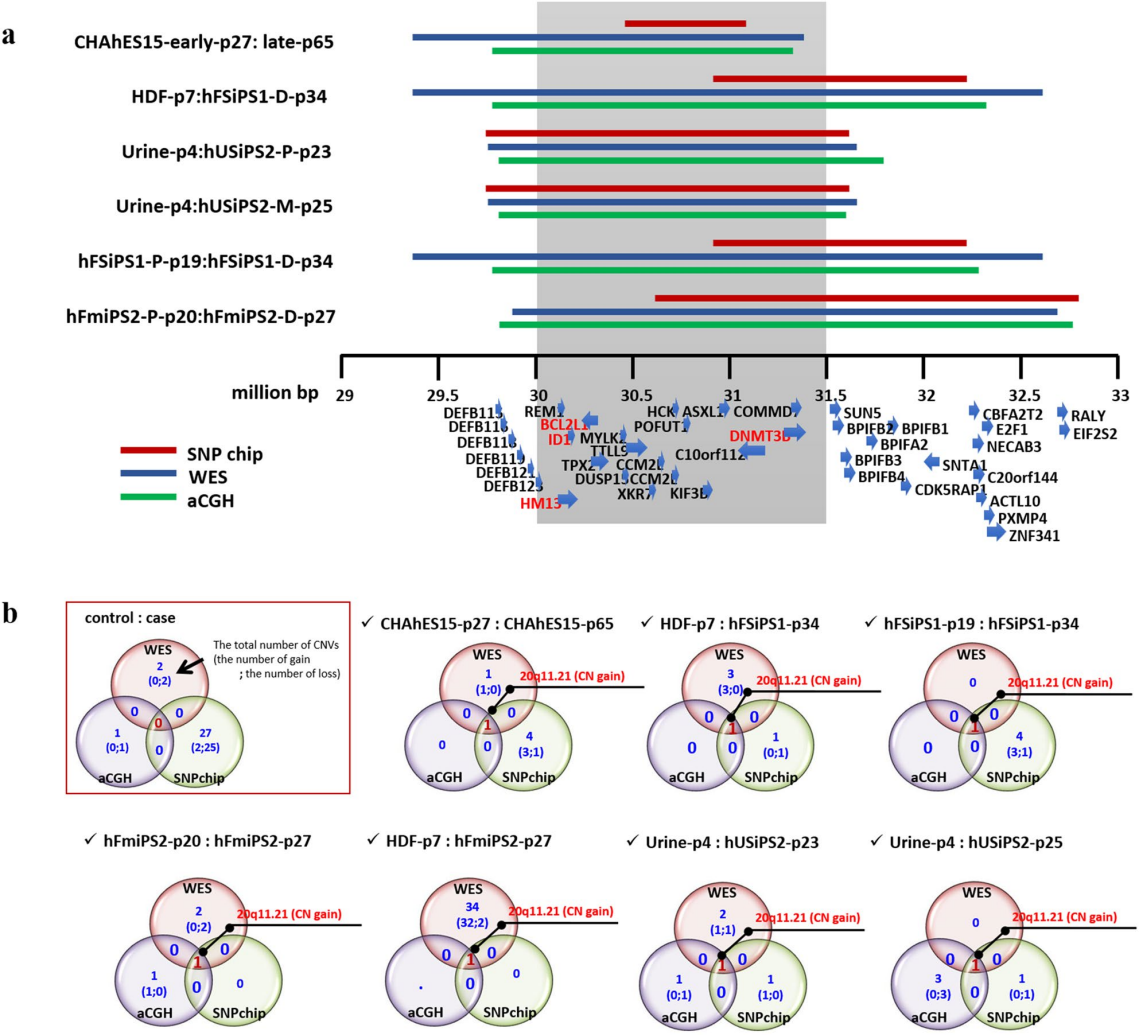
Details of the region of recurrent CNV gain at 20q11.21 with WES, SNP array, and aCGH analyses. (**a**) Details of the region of recurrent CNV gain at 20q11.21. Red, blue, and green bars indicate the breakpoint of the CNV gain at 20q11.21 using SNP array, WES, and aCGH analysis, respectively. The core range, including the candidate *BCL2L1*, *ID1*, *DNMT3B,* and *HM13* genes with strong selective advantages, is shaded in grey. This analysis was performed for control-case paired samples, showing the control: case cell lines on the left. The genes in the region of the CNV gain are specified with strand directions at the bottom. (**b**) Venn diagrams illustrating CNVs for paired samples from three platforms. Only pairs with CNV gain at 20q11.21 were specified. The CNV results for other paired samples are illustrated in Supplementary Fig. S2. A detailed explanation of the Venn diagrams is given in the red box.

We evaluated copy number changes in hPSCs based on the Whole-exome sequencing (WES), SNP array, and aCGH data with respect to the time in culture and reprogramming method. To compare the control-case samples, we created Venn diagrams in which intersectional comparisons were identified in a 2 × 2 comparison. The CNV profiles for the hPSCs tested in this work are described in Supplementary Table S2. A CNV was considered significant when detected by at least two platforms. In the hFmiPS1 line, the CNV gain at 4q35.2 and CNV loss at 1p21.1-p13.3 were detected by three platforms, and the CNV gain at 1q21.3, which has not yet been reported, was also detected in the WES and SNP chips. Genes in these CNV regions did not include candidate genes for stemness-, differentiation, or cancer-related functions, suggesting that they may not affect stem cell characteristics (Supplementary Fig. S1b). The CNV gain at 20q11.21 was the most notable CNV event affecting the stem cell line characteristics (Fig. 1a,b, Supplementary Fig. S2).

Five of the 14 hPSC lines in this study showed recurrent CNV gain at 20q11.21. The location of the CNV gain at 20q11.21 was identified in all analytical platforms, although the breakpoints differed slightly (Fig. 1a, Supplementary Table S2). Based on previous studies showing that the *BCL2L1* gene in the amplified CNV region confers a strong selective advantage in hPSCs during culturing^8,16,21,25^, we focused on the core region including this BCL2L1 gene (which ranged from 30,000,000 to 31,500,000 bp), which drives culture adaptation of hPSCs. Recurrent CNV tends to be acquired by prolonged culture, particularly by chromosomes 1, 12, 17, and 20, as it confers a growth advantage^15,25^. We investigated whether CNV amplification occurred over a specific passage number. Of the two hESC lines, CNV gain at 20q11.21 was detected at passage 65 for CHAhES15, indicating that this line exhibited a change in copy number at 20q11.21 during prolonged culture. Overall, CNV gain at 20q11.21 was detected in three of seven cases over an extended culture period when comparing hPSCs between the early and late passages. However, the CRMiPS lines did not gain CNVs at 20q11.21, even at late passages (passages 70 and 73). This result indicated that there was no absolute passage number at which the CNV gain at 20q11.21 occurred and that the change in copy number was dependent on the environment during culture^26^.

We confirmed the presence of CNV gain at 20q11.21 with the TaqMan CNV assay and identified a positive correlation between change in copy number and expression (Supplementary Fig. S3). Eight genes—ASXL1, BCL2L1, DNMT3B, HCK, HM13, ID1, KIF3B, and PDRG1—located within the amplicon on 20q11.21 were validated with qPCR. When the Urine-p4 and hFSiPS1-P19 lines (with no CNV gain at 20q11.21) were considered controls, the copy number levels generally showed a threefold increase.

### Transcriptome analysis of hPSCs with CNV gain at 20q11.21

**Figure 2:**
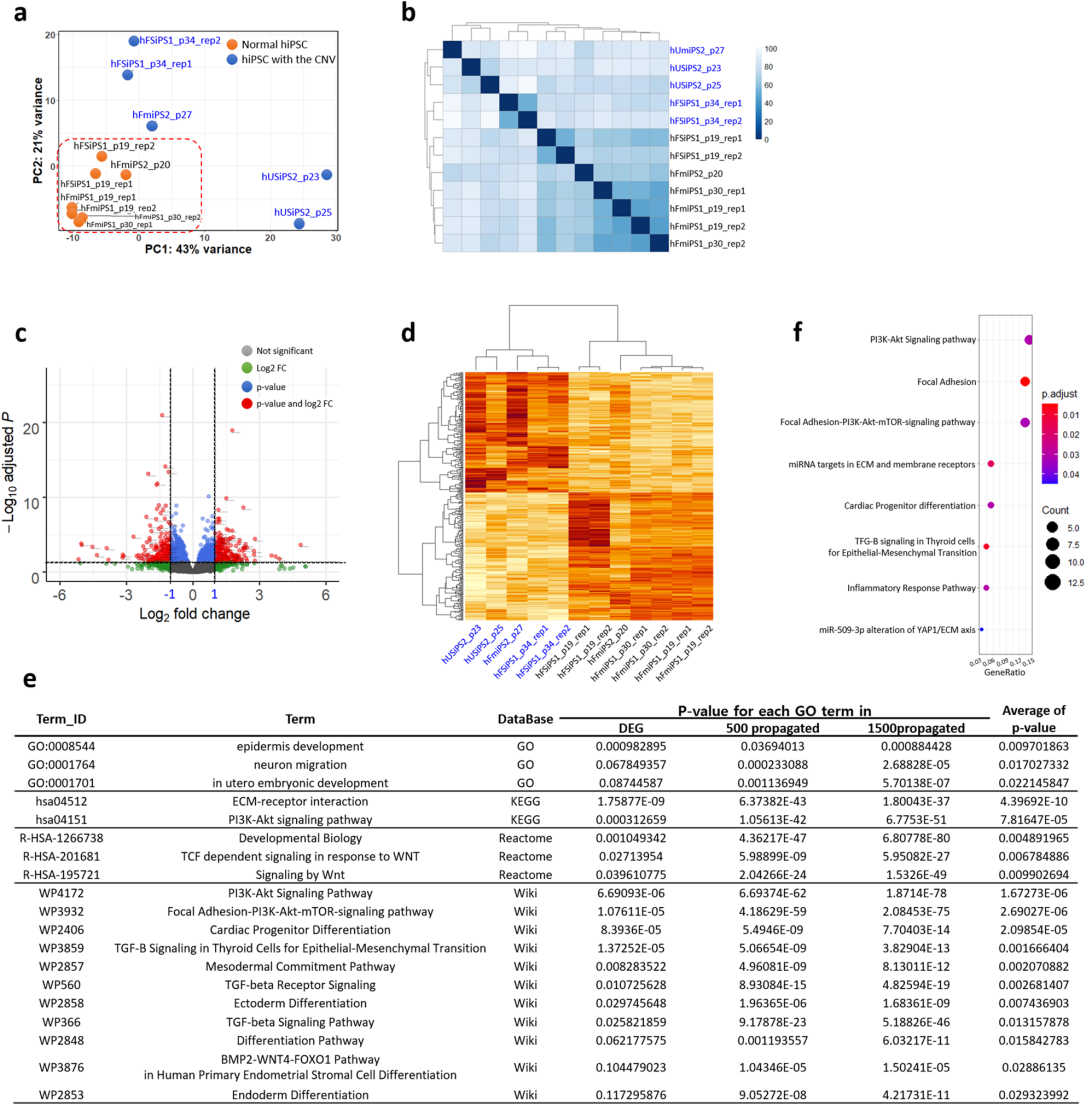
Transcriptome profiling of hPSCs with CNV gain at 20q11.21. (**a**) A principal component analysis (PCA) plot for all 12 hiPSC lines from the RNA-seq data dataset is shown. Orange and blue dots indicate normal hiPSCs and hiPSCs with CNV gain, respectively. (**b**) Sample distance matrix and unsupervised hierarchical clustering for all samples in the expression space. This matrix was constructed with the distance between samples based on normalized expression values based on the “varianceStabilizingTransformation (vst)” method. (**c**) Scatterplot of the DEGs identified in this study. Significantly up- and downregulated genes are represented by red dots. Normalized count-based analysis was performed for the control and case studies. Grey, green, blue, and red dots indicate “not significant”, genes with |log2FC|≥ 1, genes with p-value > 0.05, and genes with |log2FC|≥ 2 and p-value > 0.05, respectively. (**d**) Heatmap and unsupervised hierarchical clustering for samples based on only DEG sets. The higher the value of the log2-fold change, the darker the red colour is. (**e**) Representation of GO results for 169 downregulated genes, 500 propagated genes, and 1500 propagated genes in four different GO databases, DAVID, KEGG, Reactome, and Wikipathways. The average p-value indicates the mean p-value for each GO term derived from DEG, 500 propagated genes, and 1500 propagated genes. A full list of GO results is specified in Supplementary Table S4. (**f**) Dot plots for significant GSEA terms of down-DEGs. Dot sizes indicate GeneRatio. GeneRatio indicates the gene counts involved in each GSEA term. Adjusted p-values represented in colour gradient ranging from red to blue, corresponding to the- increasing adjusted p-values. hiPSCs with CNV gain are represented in blue. Rep1 and rep2 indicate replicates of RNA-seq for the sample.

To assess the global effects of CNV gain at 20q11.21 on hPSCs at the transcriptomic level, we measured and compared the expression levels of the hPSC lines with and without the CNV amplicon at 20q11.21 with RNA-seq. Principal component analysis (PCA) of all the hiPSC lines based on expression revealed that the expression pattern of the hiPSCs with CNV gain differed from each other, while normal hiPSCs closely resembled each other (Fig. 2a). Moreover, unsupervised hierarchical clustering and sample distance with normalized gene counts using the variance stabilizing transformation method mainly showed two groups: normal hiPSCs and hiPSCs with CNV gain (Fig. 2b).

The differentially expressed genes (DEGs) were considered significant when |log_2_FC| was ≥ 1 and the false discovery rate (FDR) was < 0.05. Supplementary Table S3 lists the significant DEGs. Of the 328 significant genes, 159 were upregulated and 169 were downregulated (Fig. 2c,d). To investigate the biological processes identified by the DEGs, we performed a comprehensive functional enrichment analysis and a gene set enrichment analysis (GSEA) based on network propagation analysis^27^. We obtained six gene sets (159 UP-DEGs, 500 and 1500 UP-DEG-cascaded genes, 169 DOWN-DEGs, 500 and 1500 DOWN-DEG-cascaded genes) and performed GSEA for these six gene sets with log_2_(FC) values for four sources (Gene Ontology (GO)^28^ biological process, KEGG^29^, Reactome^30^, and WikiPathways^31^). Then, the enriched pathways were ordered in terms of the average p-values, and the top enriched pathways are listed for down-DEGs in Fig. 2e and Supplementary Table S4. Differentiation- and development-related pathways were commonly highly ranked in the enriched pathways of DOWN-DEGs, indicating that these altered signalling pathways due to the CNV aberration at 20q11.21 affect the characteristics of hPSCs in terms of pluripotency, differentiation and development (Fig. 2e). In particular, gene set enrichment analysis (GSEA) for downregulated DEGs revealed that PI3K signalling pathways were downregulated, indicating that hiPSCs would be affected by changes in the PI3K signalling pathways due to copy number changes at 20q11.21 (Fig. 2f). These results indicated that the CNV gain at 20q11.21 could have a negative effect on the differentiation capacity, as well as a selective advantage.

### Alteration of embryoid body (EB) cell distribution by 20q11.21 duplication at the single-cell level

Spontaneous differentiation of EB showed heterogeneity of the differentiated cell types. The distribution shift of the heterogeneous cell population for differentiated cells from hiPSCs carrying CNV aberrations could reflect the effect on differentiation propensity due to CNV amplification. To ensure heterogeneity of EBs due to CNV amplification on 20q11.21 at the single-cell level, we subjected two independent cell lines to scRNA-seq: hFSiPS3 (normal) and hFSiPS1 with CNV at 20q11.21 (abnormal) (Supplementary Fig. S4a) and hFmiPS2 at passage 22 (normal) and hFmiPS2 with CNV at 20q11.21 at passage 30 (abnormal) (Supplementary Fig. S4b). All four of these cell lines, hFSiPS3 and hFSiPS1, originated from the same primary fibroblast cell line (HDF-p7). We carried out an integrated analysis to compare these two datasets to identify cell-type specific clusters due to CNV and cell types that appeared in both datasets. These integration analyses were performed for two distinct groups as follows: Group 1 included hFSiPS3 and hFSiPS1, and Group 2 included hFmiPS2 at p22 and p30 to compare transcriptional heterogeneity and reduce line variation. This approach is focused on the difference in distribution in the integrated dataset between the control and case samples to determine the transcriptional heterogeneity of isogenic hiPSC lines due to copy number changes.

**Figure 3:**
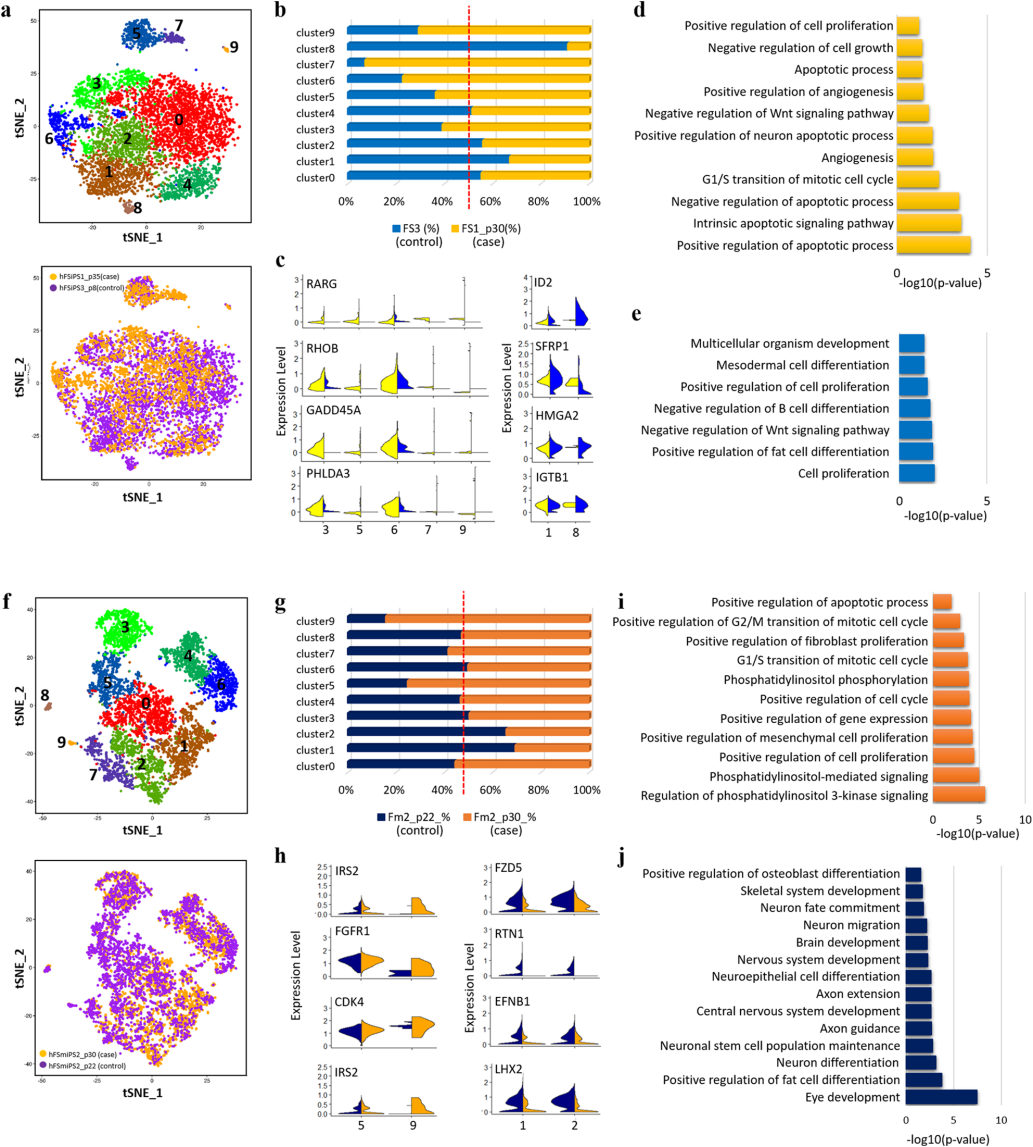
Transcriptional heterogeneity of EBs derived from hPSCs with CNV gain at 20q11.21. (**a**) tSNE plot of 6590 cells, coloured by cluster (top) and the sample origin (bottom) for hFSiPS3 (control) and hFSiPS1 (case) lines. (**b**) Fraction of cells in each cluster for the hFSiPS3 and hFSiPS1 lines. Blue bars indicate the proportion of the number of cells in each cluster of hFSiPS3, and yellow bars indicate those of hFSiPS1. (**c**) Violin plots showing the expression distribution of selected genes that are detected in higher proportions in hFSiPS1 (left, case) and in hFSiPS3 (right, control). GO results for clusters 3, 5, 6, 7, and 9 are shown in (**d**), and those for clusters 1 and 8 are shown in (**e**). (**f**) tSNE plot for 5850 cells, coloured by cluster (top) and the sample origin (bottom) for hFmiPS2 at p22 (control) and hFmiPS2 at 30 (case) lines. (**g**) Fraction of cells in each cluster for hFmiPS2 at p22 and p30. Navy bars are indicated as the proportion of the number of cells in each cluster of hFmiPS2 at p22, and orange bars are indicated as those of hFmiPS2 at p30. (**h**) Violin plots showing the expression distribution of selected genes that are detected in higher proportion in hFmiPS2 at p30 (left, case) and in hFmiPS2 at p22 (right, control). The GO results for clusters 5 and 9 are shown in (**i**), and those for clusters 1 and 2 are shown in (**j**).

The 4808 and 2790 cells in the hFSiPS3 and hFSiPS1 samples were estimated, respectively, and clustering analysis revealed 10 clusters (Fig. 3a, Supplementary Table S5, Fig. S5a). The distribution of each cluster for each sample was proportionally evaluated to estimate the relative contribution of each cell cluster per sample (Fig. 3b). Based on the expression of differential genes and GO enrichment analysis^32^, the cell population was divided into eight clusters, including Progenitor cell-0, neural cell, stem-cell like cluster, progenitor cell-1, progenitor cell-2, progenitor cell-3, mesendoderm differentiation-related cluster, and liver cell (Supplementary Fig. S6). Out of them, we identified four distinct types of progenitor cells with different gene expression patterns.

The proportion of cells in clusters 3, 5, 6, 7, and 9 was higher in the hFSiPS1 (case) line than in the hFSiPS3 (control) line (12.86% to 8.27% in cluster 3, 9.47% to 5.41% in cluster 5, 11.73% to 3.45% in cluster 6, 4.52% to 0.35% in cluster 7, and 0.96% to 0.40% in cluster 9). Cluster 3 and 6 were annotated as stem cell-like clusters while cluster 9 as liver cells. Cluster 5 as progenitor cell-2 which is mainly associated with cell cycle-related terms. Cluster 7, progenitor cell-3, were shown to be related to differentiation terms. on the other hand, the proportion of cells in clusters 1 and 8 was higher in the hFSiPS3 (control) line than in the hFSiPS1 (case) line (17.30% to 8.48% in cluster 1 and 1.80% to 0.17% in cluster 8. Genes related to apoptotic processes (RARG, RHOB, GADD45A, and PHLDA3, GO:0043065) were upregulated in clusters 3, 5, 6, 7, and 9 (Fig. 3c). GO analysis highlighted that while upregulated genes in clusters with higher fractions in the hFSiPS1 (case) lines 3, 5, 6, 7, and 9 were significantly associated with cell proliferation, cell growth, apoptosis and cell cycle-related categories (Fig. 3d), upregulated genes in clusters with higher fractions in the hFSiPS3 (control) line were significantly associated with differentiation-related categories, especially neural cell terms (Fig. 3e, Supplementary Fig. S6b). This result suggests that compared with the normal hiPS cell line, the hiPS cell line with CNV gain showed upregulated transcriptional heterogeneity related to the cell cycle, cell proliferation, and cell growth, while the normal hiPS cell line showed downregulated transcriptional heterogeneity related to differentiation compared with the hiPS cell line with CNV gain.

Additionally, 3424 and 3428 hFmiPS1 cells in the p22 and p30 samples, respectively, were estimated. Among 10 clusters calculated in the integration analysis of these two samples (Fig. 3f, Supplementary Table S6, Fig. S5b), seven distinct clusters were annotated as progenitor cell-0, neural cell, stem cell-like cluster, progenitor cell-1, progenitor cell-2, progenitor cell-3, and mesendoderm differentiation-related cluster^32^. Clusters 1 and 2 showed a higher fraction of hFmiPS2 at p22 (control): 9.28% to 21.02% in cluster 1 and 8.87% to 16.93% in cluster 2 from hFSmiPS2 at p30 (case) to p20 (control) (Fig. 3g). These two clusters were annotated as neural cell based on GO enrichment analysis (Supplementary Fig. S7) genes upregulated in clusters 1 and 2 are associated with differentiation (Fig. 3j). In addition, clusters 5 and 9 showed a higher fraction of hFmiPS2 at p30 (case) than at p22 (control) (Fig. 3g): 5.26% to 15.96% in cluster 5 and 0.20% to 1.10% in cluster 9 from hFmiPS2 at p20 (control) to p30 (case). GO analysis revealed that genes upregulated in clusters 5 and 9 are associated with cell proliferation, the cell cycle (Fig. 3i, Supplementary Fig. S7b), indicating similar results with integrated data of the hFSiPS3 and hFSiPS1 lines. In particular, gene classes associated with the PI3K signalling pathway (IRS2) and cell cycle-associated pathway (FGFR1 and CDK4) were upregulated in the EBs derived from the hPSC line with CNV amplification (Fig. 3h). Additionally, genes associated with neuron differentiation (FZD5 and RTN1, GO:0030182) and axon guidance (EFNB1 and LHX2, GO:0007411) were upregulated in the EBs derived from the normal hPSC line (Fig. 3h).

### Alteration of spontaneous differentiation by 20q11.21 duplication in vivo

We produced two and three teratomas from genetically identical iPSC sublines without duplication at 20q11.21 (hFSiPS3, control) and with duplication at 20q11.21 (hFSiPS1, case), respectively. After each teratoma was cut into several pieces, approximately half of the tissue was selected for histological analysis, while the other half was processed for RNA-seq and CNV analysis. We confirmed the differentiation potential of the teratomas according to the CNV gain at 20q11.21 at the transcriptome level. We first examined the CNV gain at 20q11.21 for teratomas originating from hFSiPS1 and hFSiPS3 (Supplementary Fig. S4c,d, respectively). CNV at 20q11.21 was detected in hFSiPS1 and not in hFSiPS3, as expected.

**Figure 4:**
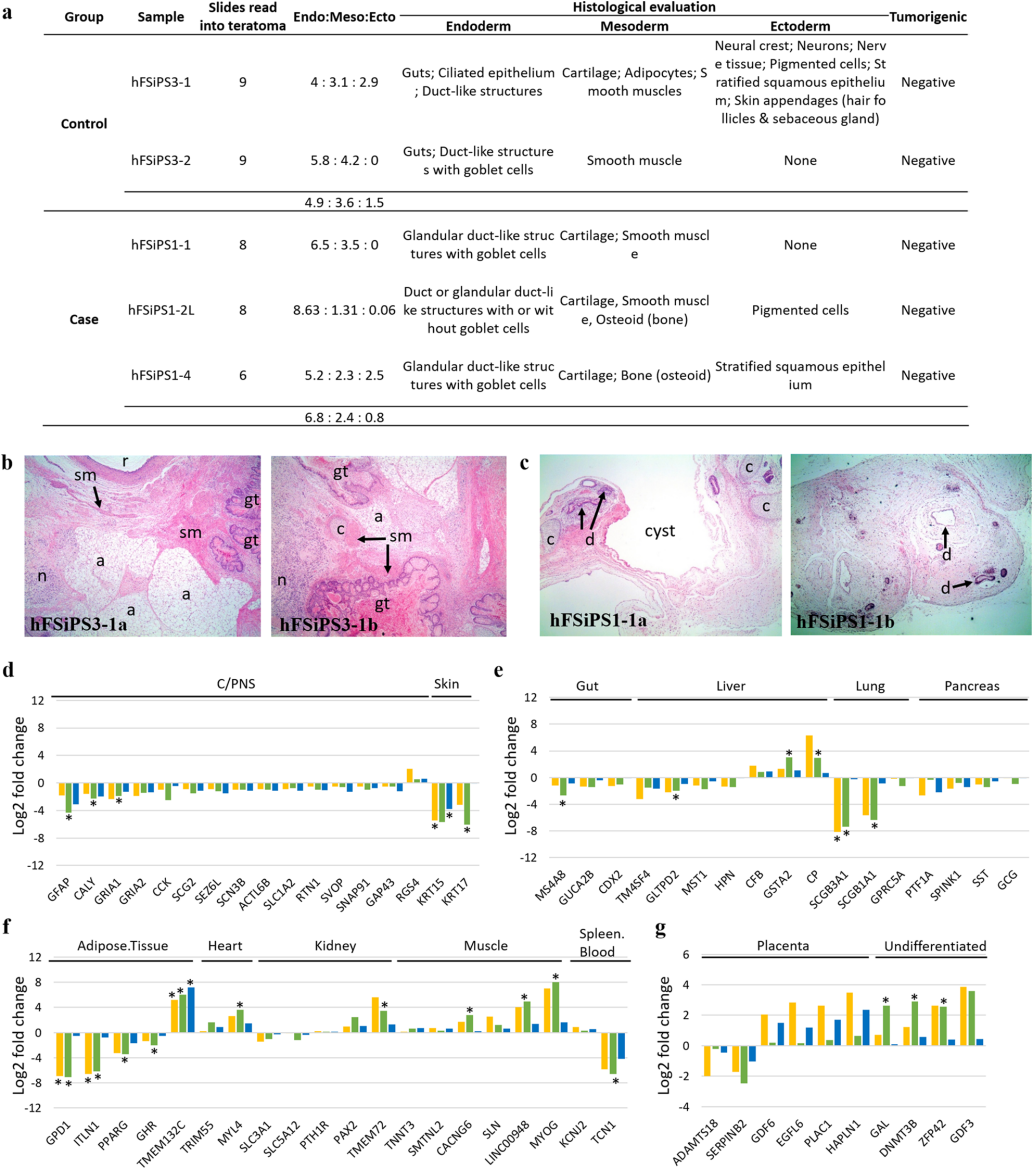
Histological evaluation and RNA-seq analysis of teratomas from hiPSCs with and without CNV gain at 20q11.21. (**a**) Summary of the tissue types recorded for individual teratoma samples. (**b**,**c**) H&E stained representative images of histological features of hFSiPS3 (**b**) and hFSiPS1 (**c**) are shown. (hFSiPS3-1a~b) Note the relatively well-differentiated tissues with three different origins showing gut (gt), cartilage (c), adipocytes (a), smooth muscle (sm), nerve tissue (n), and (r). (hFSiPS1-1a~b) A few tissue types were evident, including duct-like structures (**d**) and cartilage (**c**). Magnification, × 40 for all. Expression of ectoderm (**d**), endoderm (**e**), mesoderm (**f**), and extraembryonic (**g**), specific signatures of teratomas are shown. Yellow, green, and blue bars indicate the log2-fold changes of group A, group B, and group C, respectively. Samples in group A are only included teratomas from one hiPSC line (hFSiPS3) with or without the CNV gain. Samples in group B are included teratomas from one normal hiPSC line (hFSiPS3) and abnormal hiPSC line (hFmiPS2). These three cell lines are originated from the same fibroblast. We pooled all RNAseq data of teratomas from normal and abnormal cell lines to exclude line variation and compare non-isogenic cell lines. Detailed information for the three groups is described in Supplementary Table S8. *Indicates significance of the DEGs under adjusted p-value < 0.05. C/PNS indicates the central or peripheral nervous system. (**d**–**g**) Expression of lineage-specific signatures of teratomas.

Histological analysis of the derived teratoma sections revealed tissues identified as derivatives of the three germ layers, and no teratocarcinoma cells were observed in any section (Fig. 4a, Supplementary Table S7). Overall, for the hFSiPS3 line without duplication at 20q11.21, a median of 49% (range 43–58%) of the differentiated tissues observed were of endodermal origin, 36% (range 31–42%) were of mesodermal origin, and 15% (range 0–29%) were of ectodermal origin (Fig. 4a,b, Supplementary Fig. S8a). For the hFSiPS1 line with duplication at 20q11.21, a median of 68% (range 52–65%), 24% (range 13–35%), and 8% (range 0–25%) of the differentiated tissues were of endodermal, mesodermal, and ectodermal origin, respectively (Fig. 4a,c, Supplementary Fig. S8b). The hFSiPS1 line had a narrower ectodermal ratio than the hFSiPS3 line.

To corroborate these histological results, we also performed DEG analysis in parallel. We added RNA-seq data for 5 additional normal teratomas and 9 additional teratomas with CNV gain to remove biased results from line variation so that transcriptional profiling for a total of 7 normal teratomas and 12 teratomas with CNV gain was investigated. Transcriptome profiling was performed on three groups to consider and compare the isogenic or nonisogenic cell lines (Supplementary Table S8). The sample distance based on the expression matrix revealed that the samples were not clearly divided into two classes due to CNV amplification (Supplementary Fig. S9). To determine differential expression for lineage-specific gene signatures in the stem cell-derived teratomas at the transcriptome level, we used TeratoScore gene sets^33^ to determine the cell composition of the teratomas based on the expression level in hiPSCs with CNV gain at the transcriptional level. Among a total of 100 gene signatures provided in TeratoScore, only 54 genes were detected in our dataset. Except for only one upregulated gene, RGS4, all ectoderm-specific genes were downregulated in the teratomas with CNV gain (Fig. 4d). In particular, the expression levels of all central or peripheral nervous system (C/PNS) genes in all three cases were downregulated in the teratomas with CNV gain. In endoderm-specific gene sets, except for three genes (CFB, GSTA2, and CP) that were upregulated, all endoderm-specific genes were downregulated in all three groups (Fig. 4e). Additionally, teratomas derived from the hPSCs with CNV gain showed various expression levels of mesoderm-specific markers and differences in expression levels in the isogenic and nonisogenic cell lines (Fig. 4f, Supplementary Table S8). For example, the fold changes in GDP1 and ITLN1 by group were substantially different. Finally, most of the genes that are specific in extraembryonic lineages were upregulated in all three groups, except for only two genes (ADAMTS18 and SERPINB2), while upregulation of undifferentiated-specific markers was identified in all groups (Fig. 4g). Although the expression fold changes between the normal and abnormal hiPSCs were separately evaluated by isogenic or nonisogenic groups, the up- or downregulation of specific genes was the same. Together, teratomas originating from hPSCs with CNV gain at 20q11.21 showed a significant decrease in neural (ectodermal) and endodermal markers, while the expression levels of signatures in mesodermal lineages were similar between teratomas from the hPSCs with and without CNV amplification at 20q11.21.

### Validation of transcriptome data by quantitative RT-PCR analysis

To confirm the RNA-seq analysis, we performed experimental validation of the DEGs by RT-qPCR. Eight upregulated and five downregulated genes of selected DEGs in the transcriptome profiling of hPSCs (Fig. 2c, Supplementary Table S3) were validated (Supplementary Fig. S10a). Although the fold changes detected by qPCR were not completely consistent with those from the RNA-seq analysis, the expression change of 13 genes detected by qPCR was similar to the direction of the fold change calculated by RNA-seq. In particular, genes associated with the PI3K-Akt signalling pathway (COL6A3) and differentiation-related genes (FCN3, NR2F2, and SNAI2) were validated.

Lineage-specific markers detected from teratomas were also validated using RT-qPCR to confirm the results from RNA-seq (Fig. 4d,f). The markers included ectoderm-specific (GEAP and CALY), mesoderm-specific (TMEM132C and MYL4) and endoderm-specific (GSTA2 and CP) genes (Supplementary Fig. S10b). As described above, the expression change of six genes from qPCR was similar to the up- and downregulated fold changes calculated by RNA-seq.

## Discussion

Genomic stability is an indicator of the quality and safety of PSCs and is a key component for quality control in stem cell banks. In particular, hPSCs manufactured for clinical and therapeutic applications should undergo detailed characterization using high-resolution platforms such as next-generation sequencing and array-based platforms (e.g., SNP array and aCGH) to observe the cell status in response to unexpected risks and various environmental factors such as freezing and thawing processes^34^. Within this context, we performed detailed genomic characterizations of cells from the KSCB using advanced analytical technologies and tools to monitor genetic, transcriptomic, and epigenetic instability on a routine basis.

With regard to genomic alterations, we focused on copy number changes in hPSCs and performed a systematic analysis using three platforms. We identified the amplicon 20q11.21 as the most common CNV hotspot, occurring in 5 of the 14 cell lines examined (Fig. 1, Supplementary Fig. S1). The CNV region at 20q11.21 has not only been reported in several larger-scale studies^18,35,36^ but was also observed at the same occurrence rate in the International Stem Cell Initiative et al.^25^ project. This region includes candidate genes, such as *DNMT3B*, *ID1*, *BCL2L1*, and *HM13*, which are associated with pluripotency and antiapoptotic effects^4^. Of these genes, *BCL2L1* is a strong candidate for driving culture adaptation^21,25^. However, the functional roles of this genomic alteration have not been systematically assessed. Therefore, we established a global expression profile of normal and abnormal hPSCs with CNV gain at 20q11.21 to determine the functional in vivo and in vitro effects based on the time in culture and differentiation status.

We evaluated the global transcriptomic changes of abnormal hPSCs with CNV duplication at 20q11.21. Although there were no expression changes of the genes in the region of the CNV at 20q11.21 in the abnormal cell lines, we identified DEGs between the hPSC lines compared to the unamplified lines at 20q11.21, which showed networks with several genes in the 20q11.21 region (Fig. 2).

Network propagation analysis is a powerful network analysis to prioritize genes that may be associated with disease genetics, offering a refined approach by simultaneously considering all possible paths between genes based on random walks with restart (RWR) formation^27^. We applied this network propagation method to the DEGs and performed GSEA of these propagated gene sets to discover biological signals interacting with one another (Fig. 2f, Supplementary Table S4). We identified common and significant GSEA results from the DEGs and 500 and 1500 propagated gene sets in four different GO databases, suggesting that the copy number changes at 20q11.21 in the hiPSCs may affect their differentiation potential, which is consistent with previous reports^35,37^. Additionally, this advanced combinatorial approach from network propagation and GSEA from four distinct databases would provide in-depth transcriptional results from genetic abnormalities^27^.

We established two clear and genetically identical control lines (hFSiPS3 and hFmiPS2 at p22) without 20q11.21 duplication both before and after embryoid body formation. Because CNV aberrations in pluripotent stem cells occurred in the process of culture, it was important to secure congenic human pluripotent cell lines to exclude other possible variables that may have caused genomic abnormalities. Using a single accurate comparable group with the same genetic background, we performed both in vitro (single-cell RNA sequencing analysis of embryoid bodies) and in vivo (teratoma assay) functional analyses. This strategy allowed compensation for limitations from the number of samples and clearly validated the differentiation propensity of the CNV amplification at 20q11.21 in spontaneous differentiation experiments while avoiding other genetically originated variations.

Interestingly, despite the variability of the hiPSCs and EB differentiation, we identified several replicated results using different analyses. The PI3K/AKT signalling pathway has an essential role in the survival of human pluripotent stem cells^38^. Transcriptome profiling of hiPSCs with CNV gain revealed that genes associated with PI3K/AKT signalling pathways were significantly downregulated (Fig. 2e,f). In particular, regulation of the expression levels of the EGR1, COL6A3, SNAI2, TNC and FN1 genes, which are involved in PI3K-AKT signalling, was identified in the hPSCs with CNV amplification. These results are consistent with the scRNA-seq results of EB differentiation derived from the hiPSCs with CNV gain (hFSiPS1 and hFmiPS2 at p30), showing that the overexpressed gene set in a higher proportion of hiPSC clusters with CNV gain is related to the PI3K-AKT signalling pathway (Fig. 3i). This result suggests that CNV amplification may be associated with the cell survival of hPSCs via the PI3K-AKT signalling pathway^38,39^, implying that the main characteristics of pluripotent stem cells are altered due to the abnormalities in terms of differentiation potential, apoptosis, or proliferation, resulting from a potential network between the overexpression of Bcl-xL gene^8,21^, or malignancy via the PI3K-AKT signalling pathway. Altogether, using combinatorial approaches, we found that the CNV at 20q11.21 may affect cell fate specification or cell lineage decision in a consistent direction and is not a random disruption.

The in vivo teratoma assay revealed that the ecto/meso/endodermal ratio was changed due to amplification (Fig. 4a, Supplementary Table S7). The results of scRNA-seq of embryoid bodies also showed that the “higher” proportion of neurall cells, occurred in embryoid bodies from the normal line rather than the mutant line (Fig. 3, Supplementary Figs. S6, S7). Both results suggest that there are hurdles in ectodermal lineage commitment in hPSCs due to the CNV gain at 20q11.21. This result is consistent with a recent study that reported that CNV gain at 20q11.21 in hPSCs leads to impaired neuroectodermal differentiation^40^. Markouli et al.^40^. confirmed their results with directed differentiation of the normal and mutant cell lines harbouring the CNV amplification, showing significantly low levels of neuroectodermal markers, such as PAX6 and SOX1, while the normal and mutant lines differentiated similarly into mesodermal and endodermal lineages. This report is consistent with our scRNA-seq analysis of embryoid bodies and our teratoma assays. In addition, our work focused on the functional in vivo and in vitro consequences of CNV aberration using sophisticated bioinformatic approaches, including analysis of heterogeneous cell populations using single-cell RNA sequencing and assessment of hPSC differentiation propensity using the TeratoScore platform with bulk RNA sequencing due to genomic mutations.

Single-cell RNA sequencing allows exploration of cell-to-cell variation and heterogeneity in stem cell populations, characterization of the features of heterogeneous cell populations^41^, and identification of fundamental evidence regarding the differentiation mechanism of stem cells^42^. Our study focused on a variety of differentiated cell types in normal and mutant lines due to CNV gain, which resulted in heterogeneous cell populations and determined cell fate or further differentiation. This strategy allowed us to evaluate the functional consequences of differentiation and cell commitment, which are the most important characteristics of pluripotent stem cells. Therefore, this detailed approach at the single-cell level was suitable for our study.

It is important to effectively assess genetic variants to distinguish between problematic and harmless changes. CNV gain at 20q11.21 is a recurrent mutation that has been repeatedly reported in human cancer cells and hPSC lines and leads to increased migratory capacities and aberrant differentiation propensities^43,44^. Moreover, patients with the 20q11.21 duplication show a distinctive phenotype, including metopic suture, developmental delay, and short hands, due to the effects of the *ASXL1* gene^45^. Based on these observations, the CNV gain at 20q11.21 might result in altered clinical features, especially developmental delays or cancer generation, by inhibiting differentiation into specific lineages.

Detecting the genetic mosaicism of hPSCs is a key step in the use of cell lines in various applications. Baker et al.^37^ revealed that the sensitivity of alternative methods, such as the qPCR assay, is approximately 10–15% for copy number changes. Additionally, comparative genomic hybridization (CGH) or SNP chip platforms were estimated to have a sensitivity of 8–20% in detecting mosaicism^46–48^, and the sensitivity of e-karyotyping was estimated to be approximately 30%^49^. These results indicate that methods to detect mosaicism in cultured cells have limitations with regard to sensitivity. To rule out some possible effects from the mosaic cell lines, we considered only common CNVs detected in three different platforms (WES, aCGH, and SNP array) to accurately detect CNVs while removing false-positive CNVs. This approach could be an alternative to reliably detect CNVs at the genome-wide level. Moreover, to overcome these technical limitations, such as sensitivity, researchers must develop a rapid screening system to detect CNV during the early stage of hiPSC reprogramming or after changes to the culture environment to detect small proportions of abnormal cells.

In conclusion, our results provide novel insights into the molecular mechanism of related phenotypes, as well as the development of the differentiation system, especially in ectodermal lineages. Moreover, this study highlights the necessity of accurate global analysis of CNV gain at 20q11.21 over culture time and during reprogramming of hPSCs on a routine basis to eliminate hPSCs carrying this mutation for cell-based clinical applications. Therefore, the CNV gain at 20q11.21 should be assessed for its effects in cell therapy studies and clinical applications.

## Methods

### Cell lines

We obtained hPSC samples from the KSCB, including four hESCs, 10 hiPSCs, and two primary cell lines, which we used for genomic profiling (Fig. 1a). Cell lines were maintained on an STO mouse embryonic fibroblast feeder layer and transferred onto Matrigel (BD Biosciences, NJ, USA). Each cell line was studied over an extended culture period. The hiPSCs were reprogrammed from primary cells as described previously^22–24^. The hiPSC lines were derived from two types of primary cells, fibroblasts and urine cells, and reprogramming was induced with Sendai virus and modified mRNA. The cell lines were studied with respect to the reprogramming method and time in culture to identify the effects of hPSCs on genomic stability. Genomic DNA and mRNA were prepared using DNA and RNA kits, respectively (Qiagen, Germany). This study was reviewed and approved by the institutional review board of Korea Centers for Disease Control and Prevention (20170306PA). All related experiments and methods were performed in accordance with the relevant guidelines and regulations.

### Study design and analysis

The CNV analysis was performed with two array-based platforms, SNP array and aCGH, and one sequencing-based platform, WES, and then integrated. These array- and sequencing-based approaches were adopted to obtain a stringent CNV callset at a genome-wide level. For determination of the differentially expressed genes between the cell lines with or without CNV gain at 20q11.21, transcriptome analysis with RNA-seq was performed (Supplementary Fig. S1b). EB formation was evaluated at day 12 for changes caused by CNV gain at 20q11.21. Functional analysis in vivo and in vitro was also performed by teratoma assay and single-cell RNA sequencing of EBs.

We established an analytical workflow to evaluate the CNV and expression patterns of hPSCs at the genomic and transcriptomic levels (Supplementary Fig. S1). Although the start and end points of the CNV differed slightly between platforms, the CNV regions were considered to be the same when all regions were included. The resulting CNV was identified to determine whether it included stemness-, differentiation-, and cancer-related markers in genes that affected the genomic stability of hPSCs. In addition, a count-based transcriptome analysis was performed to measure how the altered expression due to the CNV gain on 20q11.21 contributed to stem cell biology.

### aCGH data processing for the CNV analysis of hPSCs

Array comparative genomic hybridization (aCGH) assays were performed on the Agilent SurePrint G3 Human 2 × 400 k platform, and downstream analyses were performed using Agilent Genomic Workbench software with the ADM-2 algorithm at a default threshold of 6. The ADM-2 algorithm, provided by Agilent Technologies, determined the change point that maximized the *t*-test statistic by comparing the average values between the change point and 0^50^. The raw CNV results from all three platforms were curated manually to exclude false positives (Supplementary Fig. S1b).

### SNP chip data processing for the CNV analysis of hPSCs

SNPs were genotyped on an Illumina Omni2.5 Exome BeadChip, which interrogated 2,608,742 SNP probes across the human genome. Genomic DNA (1 µg input) was amplified and labelled according to the manufacturer’s protocols (Illumina). After genotyping and CNV calls were extracted through the genotyping module in Illumina GenomeStudio software, PennCNV^51^ (https://penncnv.openbioinformatics.org) was used to evaluate the CNV based on a hidden Markov model algorithm (Supplementary Fig. S1b). The genotype and locations of the CNVs were based on the human genome assembly hg19. For CNV calls, GC correction, a 5-kilobase (kb) size cut-off value, and a minimum of five markers were used as the analysis filtering conditions. This study considered only CNVs in exonic regions and used the UCSC (hg19) database to identify genes within the CNV areas. Manual inspection was used to filter out false positives. All CNVs spanning centromeric regions and the X and Y chromosomes were considered false positives and thus excluded.

CNV analysis using additional SNP chips was also performed on an Affymetrix CytoScan HD (Affymetrix). Genomic DNA (1 µg input) was amplified and labelled according to the manufacturer’s protocols. For CNV analysis, the raw data were analysed with Chromosome Analysis Suite (ChAS) v3.2 (Affymetrix). CNV segments exceeding 100 kbp and 25 marker counts were included in the downstream analysis.

### Whole-exome sequencing (WES) data processing for CNV analysis of hPSCs

For WES, exome regions were captured with a SureSelectXT Human All Exon V4 + UTR or V5 + UTR kit (Agilent, Seoul, Korea). Paired-end (100-bp) reads were produced, and the captured libraries were sequenced on an Illumina HiSeq 2500 system according to the manufacturer’s protocols (Illumina, CA, USA). The sequences were aligned using the BWA-mem algorithm^52^ to generate .bam files as input. A CNV kit was used to evaluate CNV in WES data under default settings^53^ (Supplementary Fig. S1b). CNV results with a call probability < 0.9 and |log_2_ratio|< 0.5 were removed. The X and Y chromosomes were also removed due to the complexity of reliably determining the CNV.

### RNA-seq data processing

mRNA-seq libraries were prepared with the Illumina TruSeq RNA Sample Preparation Kit. We sequenced 100-nt paired-end stranded reads on an Illumina HiSeq 2500 system (Illumina) according to the manufacturer’s protocols. The adapter sequence and low-quality reads were trimmed by Trimmomatic 0.36^54^ (Supplementary Fig. S1b). The reads were mapped to the reference human genome (GRCh38, hg38) using the STAR 2.5.2b aligner^55^ (https://github.com/alexdobin/STAR/). The alignment was performed with a two-pass approach by applying splice junctions detected in the first alignment run to the second alignment. A splice junction database was built from the Ensembl database (GRCh 38). After quantification of the gene-based expression counts with HTSeq-count software^56^, 16,780 protein-coding genes with more than 5 mapped reads in ≥ 1 sample were selected, and read counts were normalized by the relative log expression (RLE). The DESeq package^57^ was used to evaluate the differential expression values based on the negative binomial test.

Network propagation analysis^27^ investigated the UP- or DOWN-DEG cascaded genes. The diffusr R package (https://cran.r-project.org/package=diffusr) considered UP- or DOWN DEGs as seed genes and propagated the influence of each of the seed genes to the other genes on a protein–protein interaction network, Pathway Commons^58^. Then, the top 500 and 1500 influenced genes were selected for each of the UP- or DOWN-cascaded DEGs. Significant DEGs with |log2FC|≥ 1 and p-value < 0.055 were selected. For the six gene sets (159 UP-DEGs, 500 and 1500 UP-DEG-cascaded genes, and 169 DOWN-DEGs, 500 and 1500 DOWN-DEG-cascaded genes), we performed functional enrichment tests for GO^28^ and KEGG^29^ pathways using the DAVID^59^ website and for Reactome^30^ and WikiPathways^31^ using the Enrichr^60^ website. In addition, for all genes with log_2_(avg.Exp_CNV_/avg. Exp_Ctrl_) values with pseudo count 1, we performed GSEA for GO, KEGG, Reactome and WikiPathways using the WebGestalt^61^ website. Then, the enriched pathways were ordered by the average of p-values, and the top five enriched pathways were selected to be shown. Significant GSEA terms with an average p-value < 0.05 were considered for downstream analysis.

### qPCR validation

To validate the copy number of genes within the CNV gain region at 20q11.21 at the genomic DNA level, we selected eight candidate genes and performed quantitative PCR (qPCR) analysis of the genes with TaqMan CNV probes. The experiments were performed in triplicate, and RNase P was used as a negative control. The gene expression levels were measured using the CopyCaller software system v2.0 (Applied Biosystems, CA, USA). Error bars represent the standard error of the mean (SEM).

In addition, to confirm DEGs detected from transcriptome profiling for hiPSCs and teratomas, 8 upregulated and 5 downregulated genes were selected and validated by RT-qPCR experiments with SYBR probes. Six of the genes significantly detected in the TeratoScore signature gene set^33^ were also validated. GAPDH was used as an endogenous reference gene to normalize the expression level. Relative gene expression levels were calculated by the ${2}^{-\Delta \Delta {C}_{t}}$ method.

### Transcriptome analysis via single-cell RNA sequencing

At day 12, four EBs derived from the hFSiPS3 line, the hFSiPS1 line, and the hFmiPS2 lines at passage 20 and passage 30 were prepared for single-cell RNA sequencing. The EBs were collected from the plate and treated with 0.25% trypsin–EDTA at 37 °C for 5 min and then carefully washed with Dulbecco’s phosphate-buffered saline (DPBS) without Ca^2+^ or Mg^2+^. Next, the EBs were dissociated into single-cell suspensions using EB culture medium. Viable single cells were loaded in the GemCode Instrument (10X Genomics, CA, USA) to construct single-cell barcoded droplets using the Chromium Single Cell 3′ v2 Library according to the manufacturer’s protocol. The libraries were constructed on the Illumina HiSeq 4000 system. The Chromium Platform (10X Genomics) was used to sequence 2790EB cells from hFSiPS1, 4808 from hFSiPS3, 3424 from hFmiPS2 at p22 and 3428 from hFmiPS2 at p30.

The single-cell RNA sequencing data were preprocessed using Cell Ranger 2.1.1 Single Cell Analysis pipelines (10X Genomics). Raw FASTQ files were mapped to the human reference genome (hg38, GRCh 38) using the STAR aligner^55^. Chromium cellular barcodes were used to construct gene-barcode matrices. Filtered gene-barcode matrices, including only cellular barcodes in MEX format, were applied for downstream analysis.

Two sets of scRNA-seq expression data were analysed with the Seurat v3.0.0 R package^62^. We performed comparative analysis using the Seurat integration procedure^61^ to identify common and distinct cell types between hPSCs with and without CNV gain at 20q11.21. After creating the Seurat object based on feature expression matrices of all four EBs, we filtered out cells with unique gene counts (nFeature_RNA) less than 200 and more than 6000 and considered cells with less than 7.5% of the percentage of mitochondrial gene expression in a cell. The resulting data were normalized using the “NormalizeData” method, which normalizes the expression counts based on the total counts. We identified features that were outliers on a ‘mean variability plot’ using the ‘vst’ method with the top 2000 most variable features. Then, the single-cell data were integrated with the log-transformed single-cell expression data after finding the integration anchors using the ‘FindIntegrationAnchors’ function.

The integration analysis was performed in 2 groups, the hFSiPS3 (control) and hFSiPS1 (case) samples and the hFmiPS2 at p22 (control) and hFmiPS2 at p30 (case) samples. We scaled and centred the gene expression datasets and then used the scaled z-score residuals for principal component analysis (PCA). T-distributed stochastic neighbour embedding (tSNE) dimensionality reduction based on statistically significant principle components was performed. Shared nearest neighbour (SNN) graphs were constructed by calculating the neighbourhood overlap (Jaccard index) between every cell and its k-nearest neighbours to identify clusters of cells. A phylogenetic tree was built based on the distance matrix of clusters. Differentially expressed genes (DEGs) among clusters were calculated to identify cell type marker genes. The DEGs with an adjusted *p*-value ≤ 0.05 and log fold change ≥ 0.1 were considered in each cluster (Supplementary Tables S5, S6). The composition between clusters in the control and case samples was calculated as follows:

1 $$\frac{\text{the \,number \,of \,cells\, in\, each\, cluster}}{\text{the \,total \,number \,of\, cells\, in\, each\, cell \,line}}$$

Upregulated genes in clusters with different proportions between the control and case samples were subjected to Gene Ontology (GO) using DAVID^63^. The cell cluster were annotated based on the results from Han et al.^32^, as they analysed the scRNA-seq data from EB derived from H9, naïve-like H9 and primed H9, similar to our dataset.

### Teratoma formation in vivo

The hFSiPS3 and hFSiPS1 lines, with and without CNV gain at 20q11.21, were suspended at 1 × 10^6^ in 100 μL of phosphate-buffered saline (PBS) mixed with 50 μL of Matrigel (BD Biosciences) and injected subcutaneously into NOD/SCID (NOD. CB17-Prkdscid/J) immunodeficient mice (The Jackson Laboratory, ME, USA). Tumour growth was monitored weekly, and the mice were sacrificed after 12 weeks. After tumour resections were performed, half of the tumour tissue was placed in PBS containing 4% formalin, and the other half was used for CNV and transcriptome analysis. Genomic DNA and total RNA from teratoma tissue were prepared using SpectraFluor Plus Multifunction Fluorescence (Tecan, Switzerland) and mirVana miRNA Isolation kits (Thermo Fisher, MA, USA), respectively. At least two different teratoma blocks were sampled from each PSC line injected. For histological examination, six to nine serial sections from each teratoma block were stained with haematoxylin and eosin (H&E). All slides were examined by a pathologist who estimated the degree of differentiation in tissues derived from all three germ layers.

### Evaluation of the expression level of the TeratoScore gene set

Raw RNA-seq data were prepared for the teratomas, including 7 normal teratomas and 12 teratomas with CNV gain, as described above. Only teratoma samples with CNV gain, including the BCL2L1 gene, were analysed. The resulting reads were aligned to the human reference genome (hg38, GRCh 38) using the STAR aligner with a two-pass approach^55^. After normalization of the expression value using the regularized log transformation (RLD) method, we used a 110-TeratoScore gene set^33^ of tissue-specific genes representing the three germ layers and extraembryonic membranes, based on a previous report by ISCI^64^. The expression levels for the 110 TeratoScore gene set of tissue-specific genes were calculated for teratomas from cell lines with CNV gain at 20q11.21 compared to those without CNV gain.

## Supplementary information

Supplementary Information.

Supplementary Table S2.

Supplementary Table S3.

Supplementary Table S4.

Supplementary Table S5.

Supplementary Table S6.

Supplementary Table S7.

## Data Availability

The raw data from the SNP chip and RNA sequences used in this study were deposited in the Clinical & Omics Data Archive (CODA, https://coda.nih.go.kr) under accession numbers R001854 and R001794, respectively. The cell lines used in this study are available through the KSCB Institute (https://kscr.nih.go.kr/nscb).
