# Supplementary material for: Functional in vivo and in vitro effects of 20q11.21 genetic aberrations on hPSC differentiation
Source: Sci Rep. 2020 Oct 29;10:18582. doi: 10.1038/s41598-020-75657-7 (PMC7596514; doi:10.1038/s41598-020-75657-7)
Supplement: Supplementary file 1 — Supplementary Information. [file 41598_2020_75657_MOESM1_ESM.docx]

**Supplementary Information**

**Functional in vivo and in vitro effects of 20q11.21 genetic aberrations on hPSC differentiation**

Hye-Yeong Jo^1,2^, Youngsun Lee^1^, Hongryul Ahn^3^, Hyeong-jun Han^1^, Ara Kwon^1^, Bo-Young Kim^1^, Hye-Yeong Ha^1^, Sang Cheol Kim^4^, Jung-Hyun Kim^1^, Yong-Ou Kim^1^, Sun Kim^2,5^, Soo Kyung Koo^1^* and Mi-Hyun Park^1^*

1. Division of Intractable Diseases, Center for Biomedical Sciences, Korea National Institute of Health, Korea Centers for Disease Control and Prevention, Cheongju, Republic of Korea.

2. Interdisciplinary Program in Bioinformatics, Seoul National University, Seoul, Republic of Korea.

3. Division of Data Science, University of Suwon, Hwaseong-si, Gyeonggi-do, 18323, South Korea

4. Division of Bio-Medical Informatics, Center for Genome Science, National Institute of Health, Korea Centers for Disease Control and Prevention, Cheongju 28159, Korea5. Department of Computer Science and Engineering, Seoul National University, Seoul, Republic of Korea.

^🞶^Correspondence: skkoo@nih.go.kr; mihyun4868@korea.kr


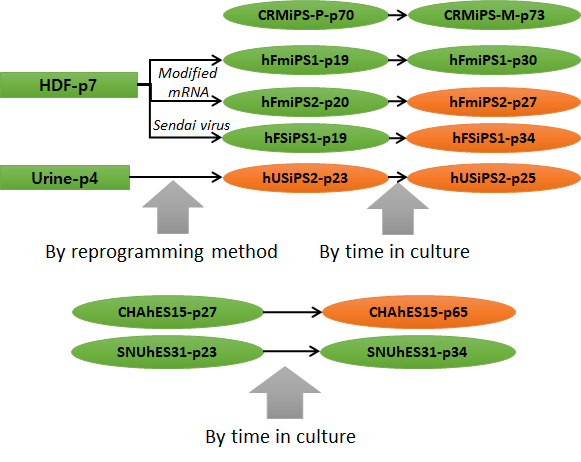
**a**


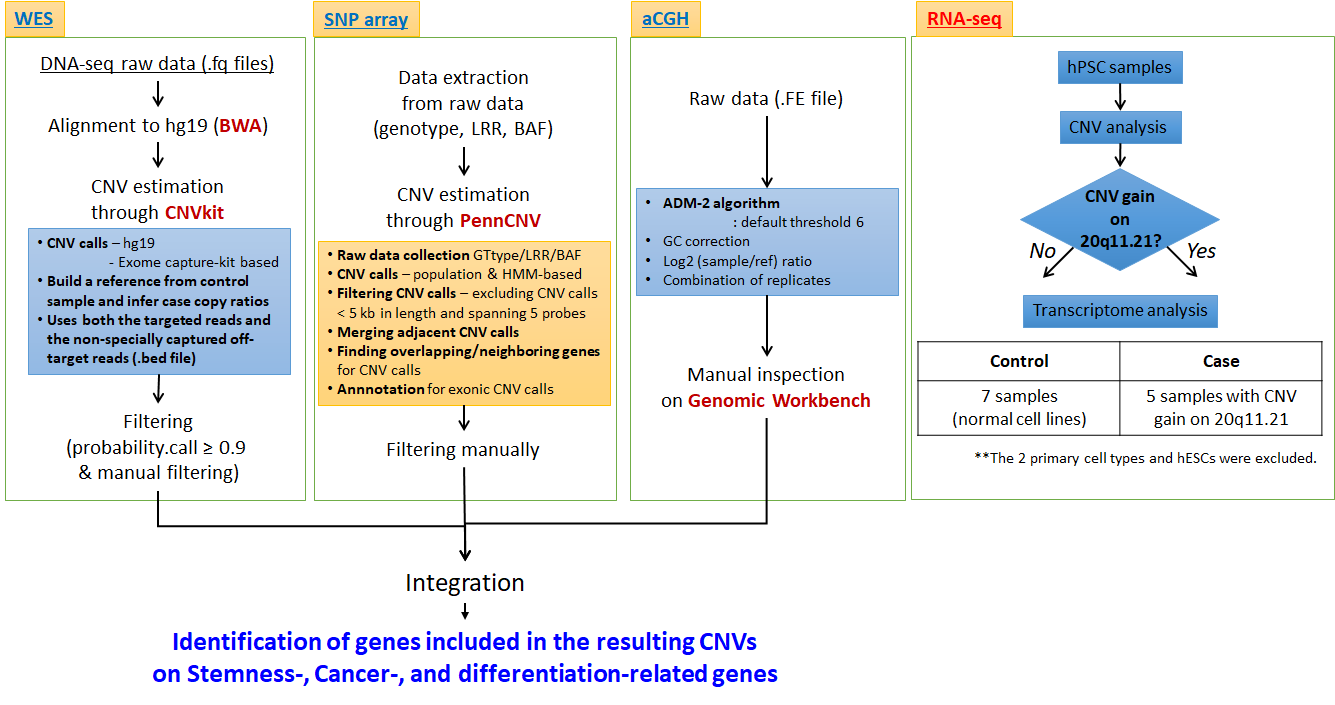
**b**

**Supplementary Figure S1. Overview of the study design and analysis strategy. (a)** Human pluripotent stem cell (hPSC) samples were analysed in this study. Copy number variation (CNV) in the two human embryonic stem cell (hESC) lines and four human-induced pluripotent stem cell (hiPSC) lines were evaluated based on reprogramming method and time in culture using whole-exome sequencing (WES), single-nucleotide polymorphism (SNP) array, and array comparative genomic hybridization (aCGH). Rectangles indicate primary cells and ovals indicate hPSC lines. Green represents cell lines with no CNV gain on 20q11.21 and orange represents those with the CNV gain on 20q11.21. **(b)** Analysis workflow of the genomic and transcriptomic data. Differentially expressed genes (DEGs) based on RNA-seq data were evaluated in hPSC lines with or without the CNV gain on 20q11.21, as a case or control. Nine samples were included as the control and five as the case.

**Supplementary Figure S2. (Related to Figure 1) Venn diagrams of the CNV in paired samples.** The number of significant CNVs between control and case samples is indicated in venn diagram. Description to explain the venn diagrams is depicted in red box. Case samples with the CNV gain are marked in red. Out of 17 pairwise comparison, 7 cases were identified the CNV gain from all three platforms including aCGH, SNP chip and WES data, described in red.

**Supplementary Figure S3.** **(Related to Figure 1) Validation of the gene dosage of the region of recurrent CNV gain on 20q11.21 with a TaqMan qPCR probe.** This evaluation was performed with eight genes in the region of the CNV gain. Cell lines with or without CNV gain on 20q11.21 are shown in orange and blue, respectively. The normal cell line, Urine-p4, is shown in black. Expression values are presented below the graph.


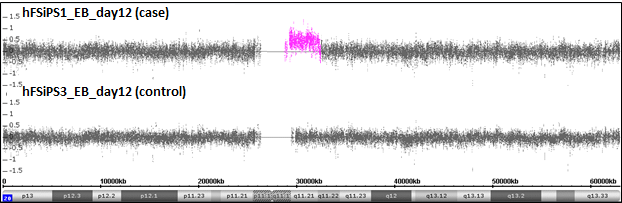
**a**


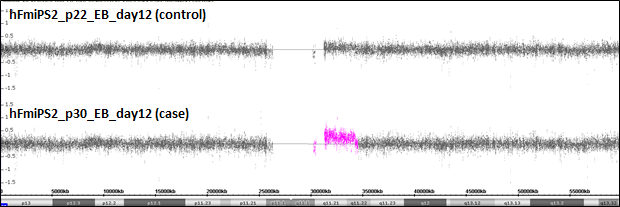
**b**


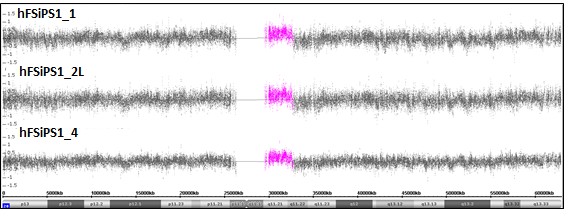
**c**


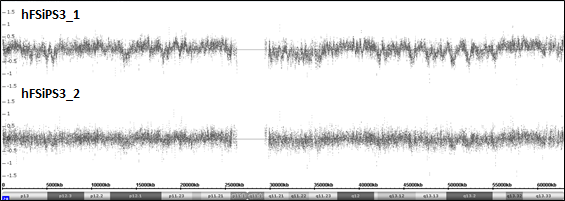
**d**

**Supplementary Figure S4. Identification of CNV gain on 20q11.21 for the hPSC samples. (Related to Figure 3 and 4)** Only the targeted CNV region on 20q11.21 is shown in colour in pink. (a) four samples performed scRNA-seq in pink (b and c) and teratomas (d and e) originating from hFSiPS1 (case) and hFSiPS3 (control), respectively. The CNV region detected were indicated in pink.


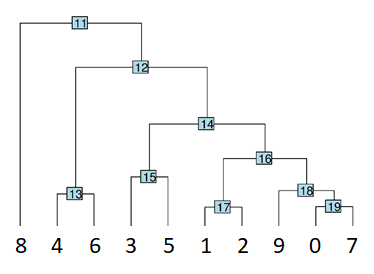
**a b**


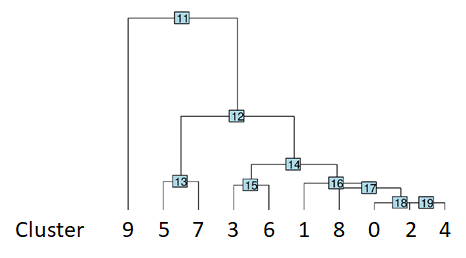


**Supplementary Figure S5. (Related to Figure 3) phylogenetic tree for each cluster in hFSiPS3 and hFSiPS1 (a) and hFSmiPS2 at passage 22 and 30 (b).** Tree was constructed based on a distance matrix from PCA space. The number below indicates the number of each cluster.


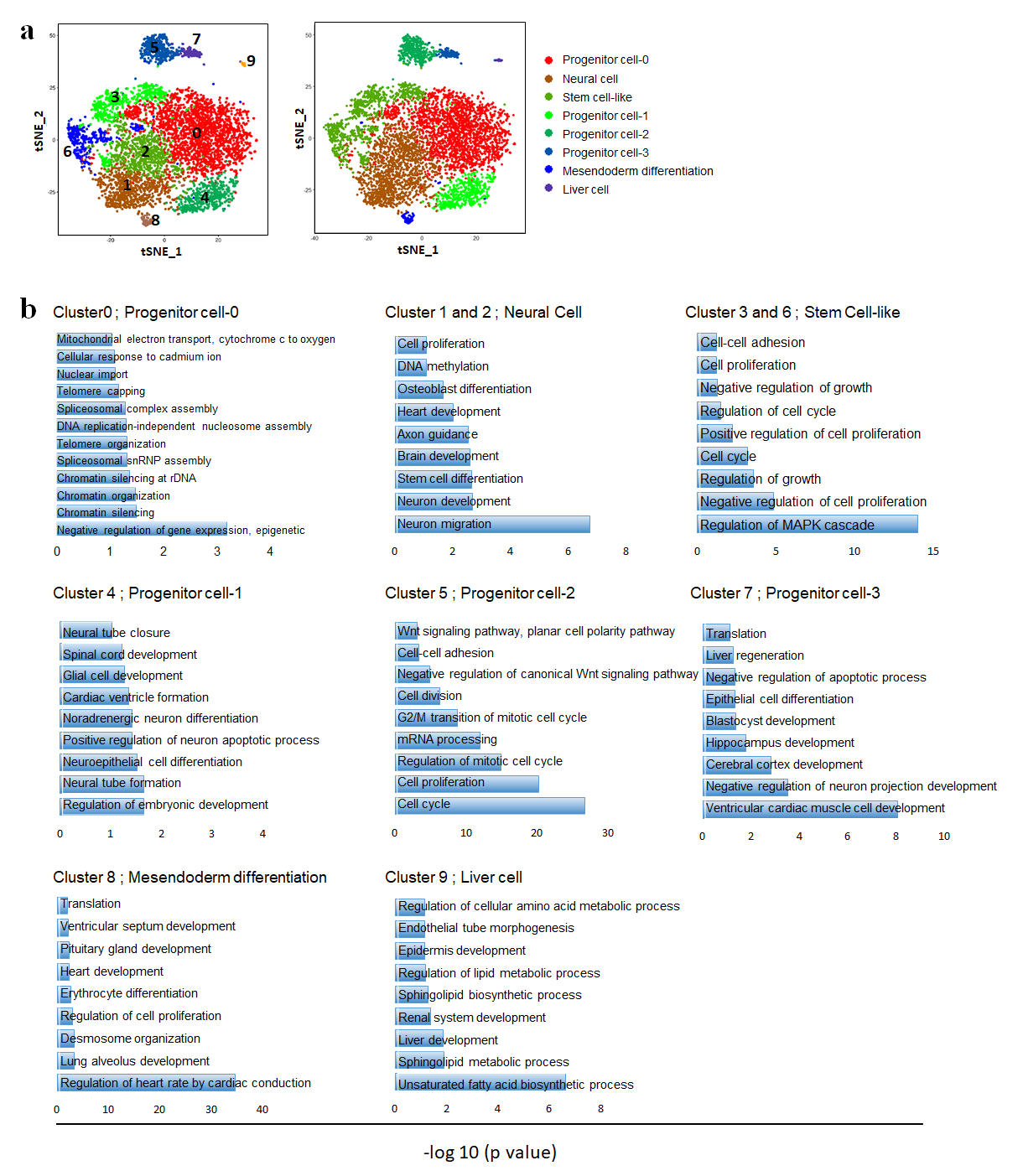


**Supplementary Figure S6. (Related to Figure 3) GO enrichment analysis of lineage progenitors in hFSiPS3 (normal) and hFSiPS1 (abnormal) samples. (a)** t-SNE plots of hFSiPS3 and hFSiPS1 samples. Eight progenitor clusters were defined, including Progenitor cell-0, neural cell, stem cell-like, progenitor cell-1, progenitor cell-2, progenitor cell-3, mesendoderm differentiation, and liver cell. **(b)** GO terms for each clusters with p-value < 0.05 are shown. P-values is shown as –log_10_(p-value).


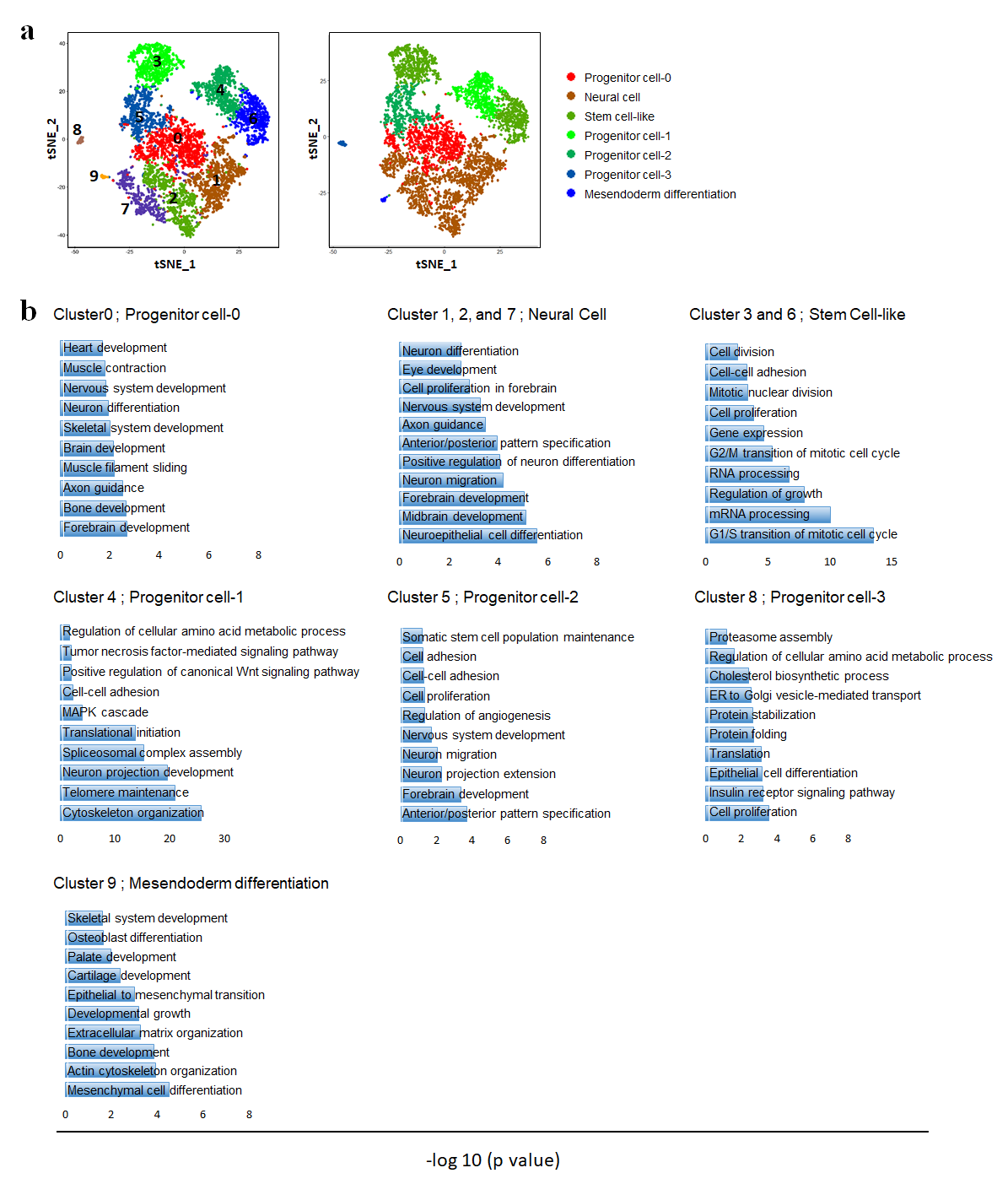
 **Supplementary Figure S7. (Related to Figure 3) GO enrichment analysis of lineage progenitors in hFmiPS2 (p22, normal) and hFmiPS2 (p30, abnormal) samples. (a)** t-SNE plots of hFmiPS2 samples at p22 and p30. Seven progenitor clusters were defined, including Progenitor cell-0, neural cell, stem cell-like, progenitor cell-1, progenitor cell-2, progenitor cell-3, and mesendoderm differentiation. **(b)** GO terms for each clusters with p-value < 0.05 are shown. P-values is shown as –log_10_(p-value).


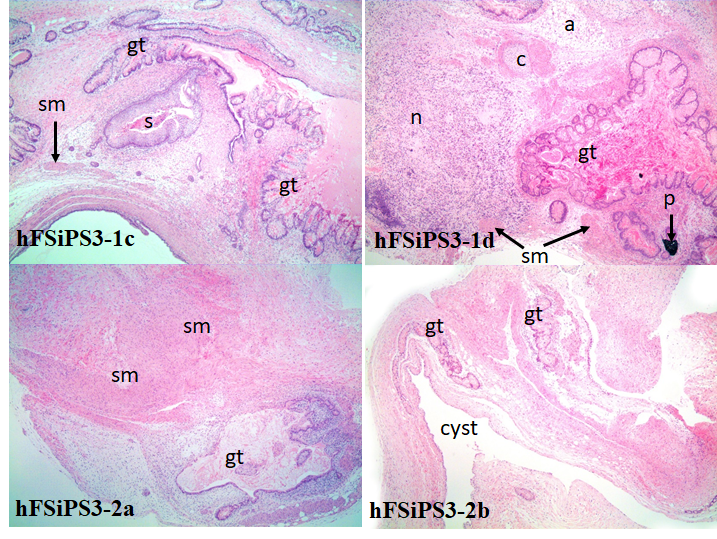
**a**


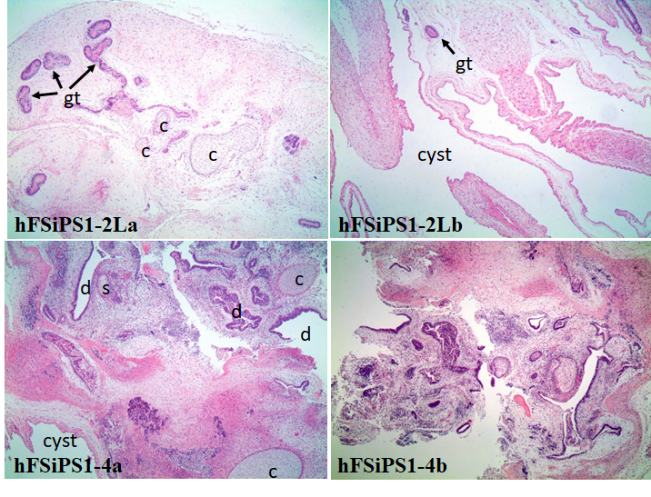
**b**

**Supplementary Figure S8. (Related to Figure 4) Histological features of terafomas derived from hFSiPS3 and hFSiPS1.** Histological features of hFSiPS3 **(a)** and hFSiPS1 **(b)**. (hFSiPS3-1c and d) Note the relatively well-differentiated tissues with three different origins showing gut (gt), cartilage (c), adipocytes (a), smooth muscle (sm), nerve tissue (n), stratified squamous epithelium (s), and pigmented cells (p). (hFSiPS3-2 a and b) Note the gut (gt) and smooth muscle (sm) with a large cyst. (H&E stain). Magnification, ×40 for all. (hFSiPS1-2La and b) Note: Only a few tissue types were noted, including gut (gt), glandular duct-like structures (d), and cartilage (c). In hFSiPS1-4, glandular duct-like structures (d), cartilage (c) and stratified squamous epithelium (s) were observed with a large cyst. (H&E stain). Magnification, ×40 for all.

**a b**


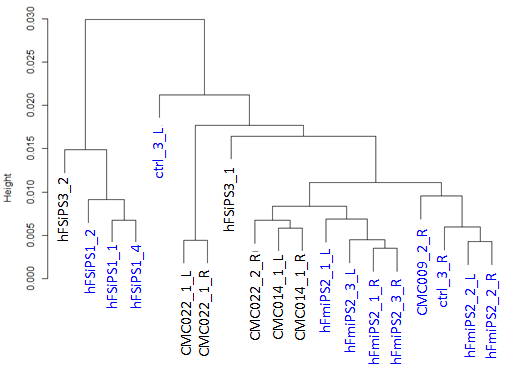
**c**

**Supplementary Figure S9. (Related to Figure 4) sample distance tree for each group performed transcriptome profiling of teratomas.** The sample distance were calculated based on complete linkage method from gene expression space.

**a**

**b**


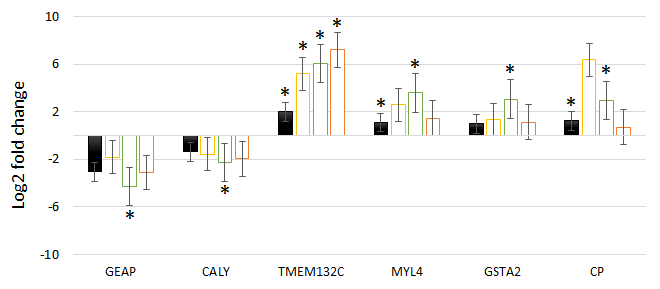


**Supplementary Figure S10. (Related to Figure 2 and 4) Validation of the DEGs with a SYBR qPCR probe.** Bars represent the log_2_ fold change in expression of each gene identified in RNA-seq from hPSCs with the CNV gain (a) and teratoma (b). Black bars represent qRT-PCR result ($2^{-\Delta\Delta C_{t}}$). White bars represent the RNA-seq results (log2 fold change). Yellow, green, and orange-squared bars represent the log fold change values in group A, B, and C, respectively, described in Supplementary Table S8. Error bars indicate the standard error. GAPDH was the internal reference. * indicates significant difference in gene expression between control and case group, respectively with qRT-PCR measured by paired t-test at p < 0.05 and RNAseq measured by DESeq2 at FDR < 0.05.

**Supplementary Tables**

**Supplementary Table S1. Sample information.** List of details for the hiPSC, hESC, and the original cell lines used in this study.

| **Cell name** | **Cell line** | **Passage** | | **Type** | **Reprogramming method** | **Primary cell** |
| --- | --- | --- | --- | --- | --- | --- |
| HDF-p7 | fibroblast | | p7 | primary cell |  |  |
| Urine-p4 | Urine | | p4 | primary cell |  |  |
| hFmiPS1-p19 | hFmiPS1 | | p19 | hiPSC | modified mRNA | HDF-p7 |
| hFmiPS1-p30 | hFmiPS1 | | p30 | hiPSC | modified mRNA | HDF-p7 |
| hFmiPS2-p20 | hFmiPS2 | | p20 | hiPSC | modified mRNA | HDF-p7 |
| hFmiPS2-p27 | hFmiPS2 | | p27 | hiPSC | modified mRNA | HDF-p7 |
| hFSiPS1-p19 | hFSiP1 | | p19 | hiPSC | Sendai virus | HDF-p7 |
| hFSiPS1-p34 | hFSiP1 | | p34 | hiPSC | Sendai virus | HDF-p7 |
| hUSiPS2-p23 | hUSiPS2 | | p23 | hiPSC | Sendai virus | Urine-p4 |
| hUSiPS2-p25 | hUSiPS2 | | p25 | hiPSC | Sendai virus | Urine-p4 |
| hFSiPS3 | hFSiPS3 | | p8 | hiPSC | Sendai virus | HDF-p7 |
| CHAhES15-p27 | CHAhES15 | | p27 | hESC |  |  |
| CHAhES15-p65 | CHAhES15 | | p65 | hESC |  |  |
| SNUhES31-p23 | SNUhES31 | | p23 | hESC |  |  |
| SNUhES31-p34 | SNUhES31 | | p34 | hESC |  |  |

**Supplementary Table S2. (Related to Figure 1 and supplementary Figure S2) CNV profiles for hPSCs examined in this study.** CNV profiles of the hPSCs examined in this study. List of the total CNV results from each platform: SNP chip, WES, and aCGH. The CNV analysis compared copy number changes between the “control” and “case” samples. The CNV results are accompanied by information pertaining to paired control/case samples, including the platform used, the chromosome, the start and end positions, length, gain or loss, and the genes included in the CNV. The CNV gain on 20q11.21, which was the focus of this study, is highlighted in yellow. Excel file available.

**Supplementary Table S3. (Related to Figure 2)** List of significant DEGs in hPSCs with the CNV gain on 20q11.21. The final genes were sorted by FDR value in ascending order. Excel file available.

**Supplementary Table S4. (Related to Figure 2)** Gene set enrichment analysis (GSEA) for propagated DEGs. GO results with average of p-value < 0.05 are shown. GO terms which is related to PI3K-Akt signaling pathways and differentiation are highlighted in yellow. Excel file available.

**Supplementary Table S5. (Related to Figure 3)** List of genes and clusters for EBs derived from hFSiPS3 and hFSiPS1. “p_val” column indicates p-value and “avg_logFC” indicates log fold-change of the average expression between the two groups. Moreover, “pct.1” and “pct.2” columns indicate the percentage of cells where the feature is detected in the cluster and other clusters, respectively. “p_val_adj” means adjusted p-value based on bonferroni correction with all features. Excel file available.

**Supplementary Table S6. (Related to Figure 3)** List of genes and clusters for EBs derived from hFmiPS2 at passage 22 and 30. Columns were described above. Excel file available.

**Supplementary Table S7. (Related to Figure 4)** Information of all individual slide sections examined from each teratoma samples. Excel file available.

**Supplementary Table S8. (Related to Figure 4)** Group information between normal and abnormal teratomas.

| **Group** | **Control** | **Case** | **Description** |
| --- | --- | --- | --- |
| Group A | hFSiPS3_1 | hFSiPS1_1 | Comparison between isogenic cell lines |
|  | hFSiPS3_2 | hFSiPS1_2 |  |
|  |  | hFSiPS1_4 |  |
|  |  | Ctrl_3_R |  |
|  |  | Ctrl_3_L |  |
| Group B | hFSiPS3_1 | hFmiPS2_1_R | Comparison between isogenic cell lines |
|  | hFSiPS3_2 | hFmiPS2_1_L |  |
|  |  | hFmiPS2_2_R |  |
|  |  | hFmiPS2_2_L |  |
|  |  | hFmiPS2_3_R |  |
|  |  | hFmiPS2_3_L |  |
| Group C | hFSiPS3_1 | hFSiPS1_1 | Pooling : comparison between non-isogenic cell lines |
|  | hFSiPS3_2 | hFSiPS1_2 |  |
|  | CMC022_1_R | hFSiPS1_4 |  |
|  | CMC022_1_L | Ctrl_3_R |  |
|  | CMC022_2_R | Ctrl_3_L |  |
|  | CMC014_1_R | hFmiPS2_1_R |  |
|  | CMC014_1_L | hFmiPS2_1_L |  |
|  |  | hFmiPS2_2_R |  |
|  |  | hFmiPS2_2_L |  |
|  |  | hFmiPS2_3_R |  |
|  |  | hFmiPS2_3_L |  |
|  |  | CMC009_2_R |  |
